# Supplementary material for: NRG1/ErbB signalling controls the dialogue between macrophages and neural crest-derived cells during zebrafish fin regeneration
Source: Nat Commun. 2021 Nov 3;12:6336. doi: 10.1038/s41467-021-26422-5 (PMC8566576; doi:10.1038/s41467-021-26422-5)
Supplement: Supplementary file 1 — Supplementary Information [file 41467_2021_26422_MOESM1_ESM.pdf]

NRG1/ErbB signalling controls the dialogue between macrophages and neural crest-derived cells during zebrafish fin regeneration

Authors

Laplace-Builhé Béryl[1], Barthelaix Audrey[1], Said Assou[1], Candice Bohaud[1], Marine Pratlong[2], Dany Severac[2], Tejedor Gautier[1], Luz-Crawford Patricia[3], Nguyen-Chi Mai[4], Mathieu Marc[1], Jorgensen Christian[1,5], Djouad Farida[1*]

Addresses

[1]IRMB, Univ Montpellier, INSERM, Montpellier, France; [2]MGX, BCM, Univ Montpellier, CNRS, INSERM, Montpellier, France; [3]Laboratorio de Inmunología Celular y Molecular, Facultad de Medicina, Universidad de los Andes, Santiago, Chile; [4]LPHI, Univ Montpellier, CNRS, Montpellier, France; [5]CHU Montpellier, Montpellier, France

Corresponding author[*]

Farida Djouad, Inserm U 1183, Hôpital Saint-Eloi, IRMB, 80 avenue Augustin Fliche, 34295 Montpellier cedex 5, France. Tel: 33 (0) 4 67 33 04 75

E-mail: farida.djouad@inserm.fr

Keywords

Zebrafish – Neural Crest-Derived Cell– Macrophage – Regeneration –Live imaging

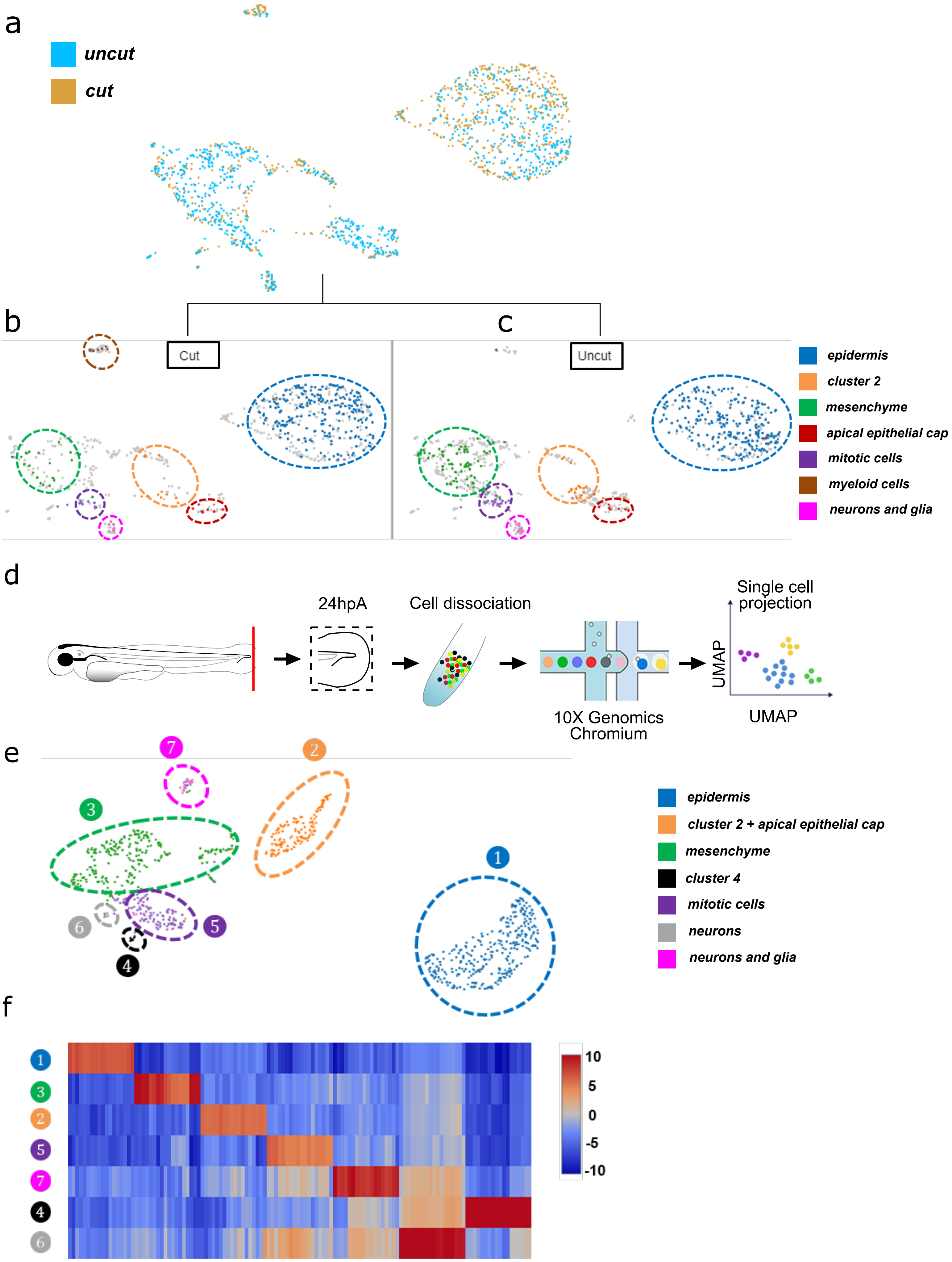
a
uncut
cut
b
Cut
c
Uncut
epidermis
cluster 2
mesenchyme
apical epithelial cap
mitotic cells
myeloid cells
neurons and glia
d
24hpA
Cell dissociation
Single cell projection
UMAP
UMAP
10X Genomics Chromium
e
7
2
3
1
6
5
4
epidermis
cluster 2 + apical epithelial cap
mesenchyme
cluster 4
mitotic cells
neurons
neurons and glia
f
1
3
2
5
7
4
6
10
5
0
-5
-10

Supplementary figure 1. **Characterization of uncut caudal cells by scRNA-seq.** **a** UMAP plots showing the aggregation using the CellRanger software between cut and uncut conditions. **b** Colour code for each cluster. **c** UMAP plots showing the scRNA-seq data for uncut and cut caudal fin fold samples after aggregation. **d** scRNA-seq experiment design. **e** UMAP plots showing the scRNA-seq data for the uncut condition. **f** Heatmap of marker gene profiles in the different clusters; red, upregulated genes, and blue, downregulated genes. **g** Colour code for each cluster.

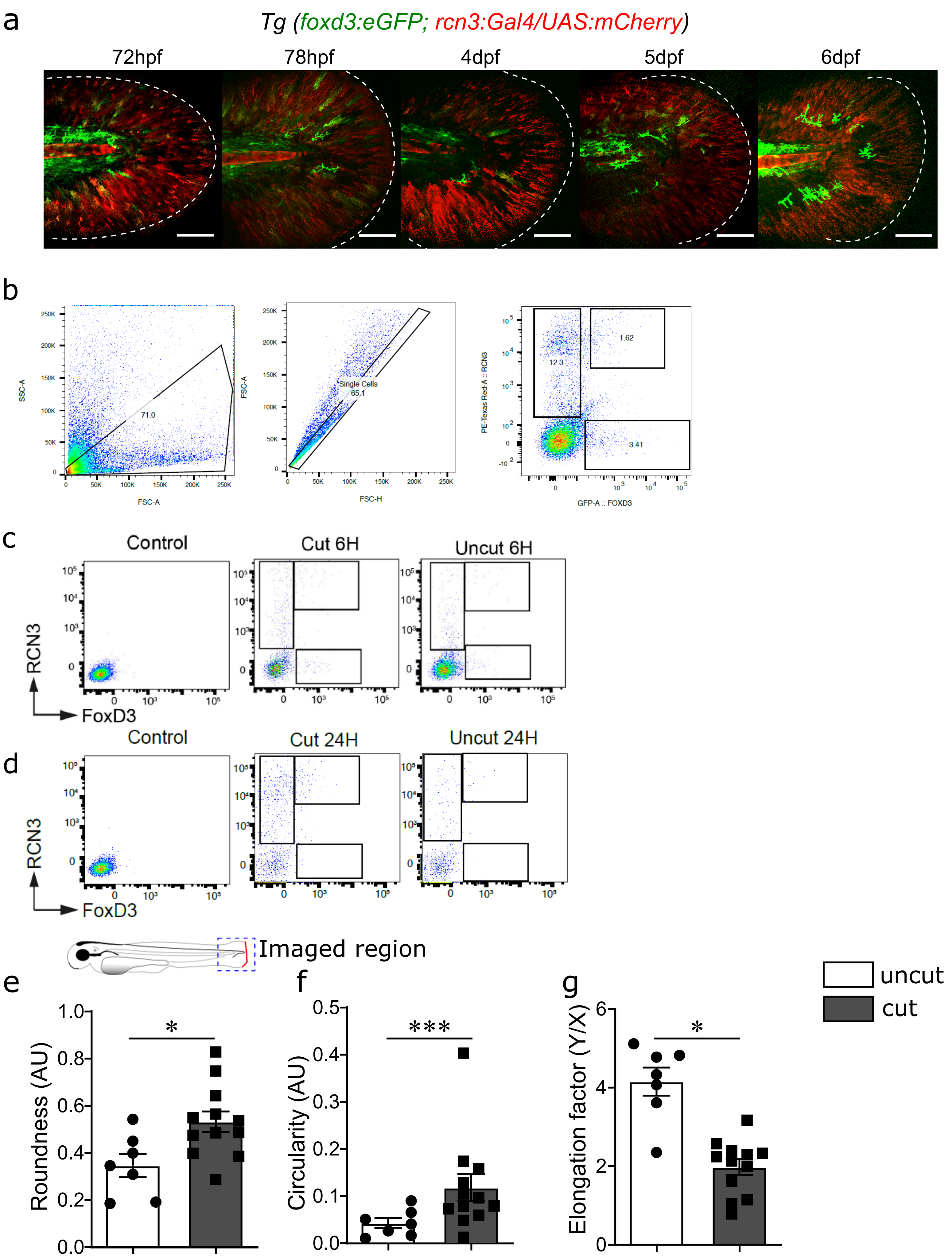
a
Tg (foxd3:eGFP; rcn3:Gal4/UAS:mCherry)
72hpf
78hpf
4dpf
5dpf
6dpf
b
71.0
SSC-A
FSC-A
Single Cells
65.1
FSC-A
FSC-H
12.3
1.62
3.41
PE-Texas Red-A :: RCN3
GFP-A :: FOXD3
c
Control
Cut 6H
Uncut 6H
RCN3
FoxD3
d
Control
Cut 24H
Uncut 24H
RCN3
FoxD3
Imaged region
e
Roundness (AU)
f
Circularity (AU)
g
Elongation factor (Y/X)
uncut
cut

Supplementary figure 2. **Frequency and behaviour of *foxd3* and *rcn3* positive cells in the regenerating fin fold. a** Representative confocal microscopy images of *Tg(foxd3:eGFP/rcn3:Gal4/UAS:mCherry)* intact larvae at different developmental time points. (Scale bars = 80µm, representative from 5 biologically independent larvae examined over 2 independent experiments). **b** Gating strategy to determine the percentage of mCherry+, eGFP+, $mCherry^{+}eGFP^{+}$ cells in dissected fin folds during caudal fin regeneration of *Tg(foxd3:eGFP/rcn3:Gal4/UAS:mCherry)* larvae. Representative FACS analysis plots of dissected fin folds from amputated *Tg(foxd3:eGFP/rcn3:Gal4/UAS:mCherry)* larvae at 6hpA **c**, and 24hpA **d** and from intact larvae at the same time as controls. **c, d, e** Cartoon showing the region imaged by confocal microscopy. **e** Roundness, **f** Circularity, and **g** Elongation factor in 3dpf *Tg(foxd3:eGFP/rcn3:Gal4/UAS:mCherry)* larvae at 6hpA were evaluated with the Fiji software. Data are the mean ± SEM, n=7 (for intact fins), and n=12 (for amputated fins) cells from 3 biologically independent larvae per groups, one-tailed Mann Whitney tests were performed, **e** p=0.0126, **f** p=0.0101, **g** p=0.0006, ***p<0.001, *p<0.05.

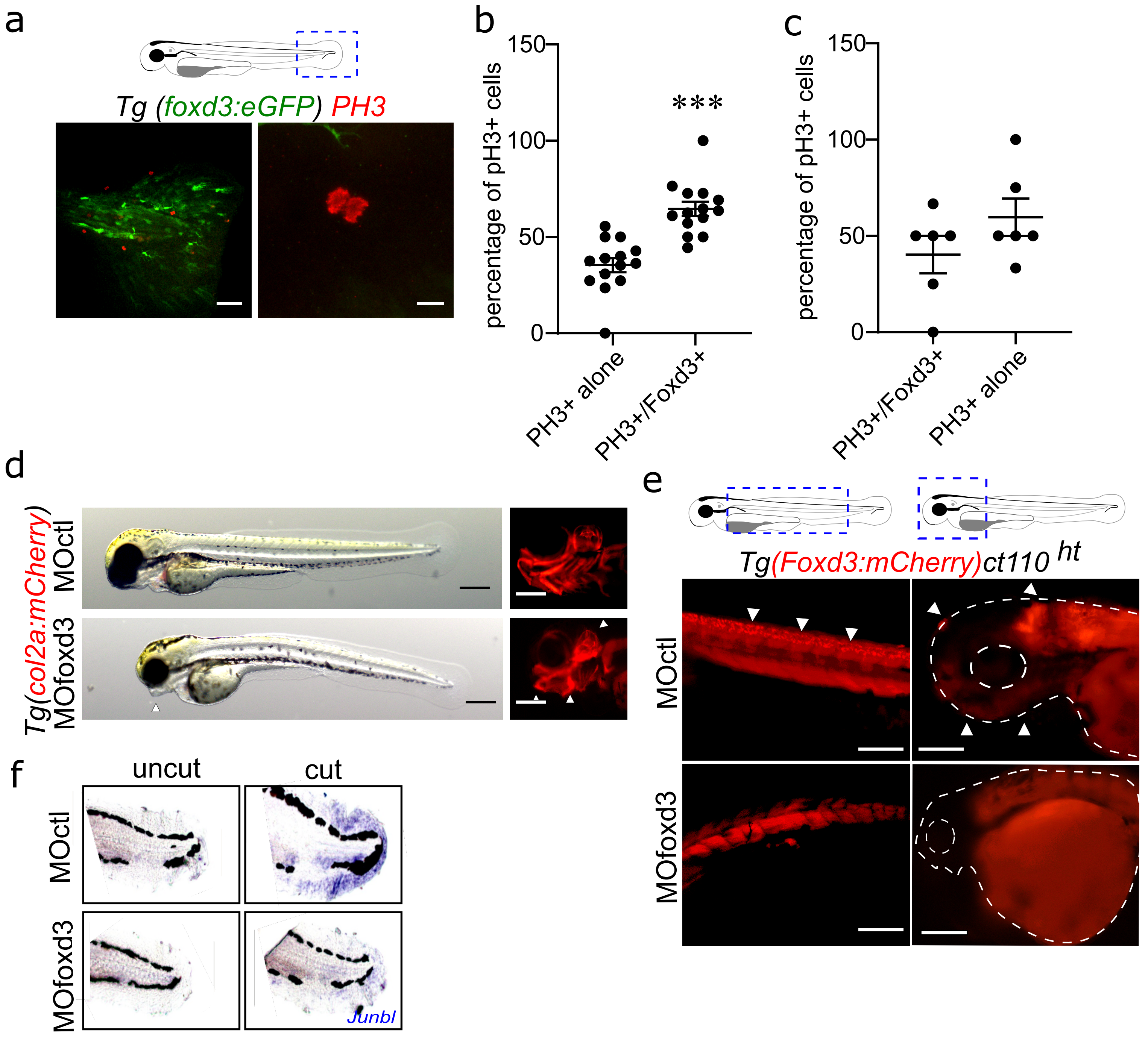
a
Tg (foxd3:eGFP) PH3
b
percentage of pH3+ cells
150
100
50
0
***
PH3+ alone
PH3+/Foxd3+
c
percentage of pH3+ cells
150
100
50
0
PH3+/Foxd3+
PH3+ alone
d
Tg(col2a:mCherry)
MOctl
MOfoxd3
e
Tg(Foxd3:mCherry)ct110 ht
MOctl
MOfoxd3
f
uncut
cut
MOctl
MOfoxd3
Junbl

Supplementary figure 3. **Blastema formation in *foxd3* deficient larvae. a** Confocal microscopy images showing PH3 labelling at 6hpA in *Tg(foxd3:eGFP)* larvae (Scale bar= 50µm), and PH3 labelled nuclei (Scale bar= 8µm, n=3 larvae). Quantification of cell proliferation in the blastema of *Tg(foxd3/UAS:eGFP)* larvae at 24hpA **b** and at the same timepoint in uninjured larvae **c**. Mitotic cells were detected using an anti-PH3 antibody. Data are the percentage of $PH3^+$ cells in contact with *foxd3*$^+$ cells ($PH3^+$/foxd3) and $PH3^+$ cells alone and, are the mean ± SEM, **b** n=14, **c** n=6, two-tailed Wilcoxon matched-pairs signed rank test were performed, **b** p= 0.0010, **c** p=0.500 ***p<0.001. **d** Transmitted light images of MO*ctl*- and MO*foxd3*-injected larvae. White arrows indicate malformation of the jaw with the Meckel's cartilage projecting abnormally ventrally (representative of 10 larvae out of 3 different experiments, scale bars=400µm). Images from fluorescent macroscopy of *Tg(col2a:mCherry)* larvae injected with MO*ctl* or MOf*oxd3* showing head cartilage abnormalities. White arrows indicate branchial arches, mandible, and otic vesicle malformations (representative of 10 larvae out of 3 different experiments, scale bars =200µm). **e** Fluorescence macroscopy images of the trunk of MO*ctl*- and MO*foxd3*-injected *Tg(foxd3:mCherry)*^{ct110} heterozygote larvae (left panels; white arrows indicate neural tube with putative NCdC) and of the head of MO*ctl*- and MO*foxd3*-injected *Tg(foxd3:mCherry)*^{ct110} heterozygote larvae (right panels; white arrows indicate pineal gland, branchial arches, and hindbrain; dotted lines show heads and eyes) (representative of 10 biologically independent larvae from one experiment were embryos were injected blindly, scale bars= 200µm). **f** *junb-l* mRNA expression by *in situ* hybridization in uncut and amputated fin fold (at 24 hpA) of control (MO*ctl*) and *foxd3* morphants (MO*foxd3*) (representative of 9 out of 12 amputated larvae and 11 out of 14 control larvae) and MO*foxd3* larvae (representative of 8 out of 13 amputated larvae and 15 out of 20 control larvae).

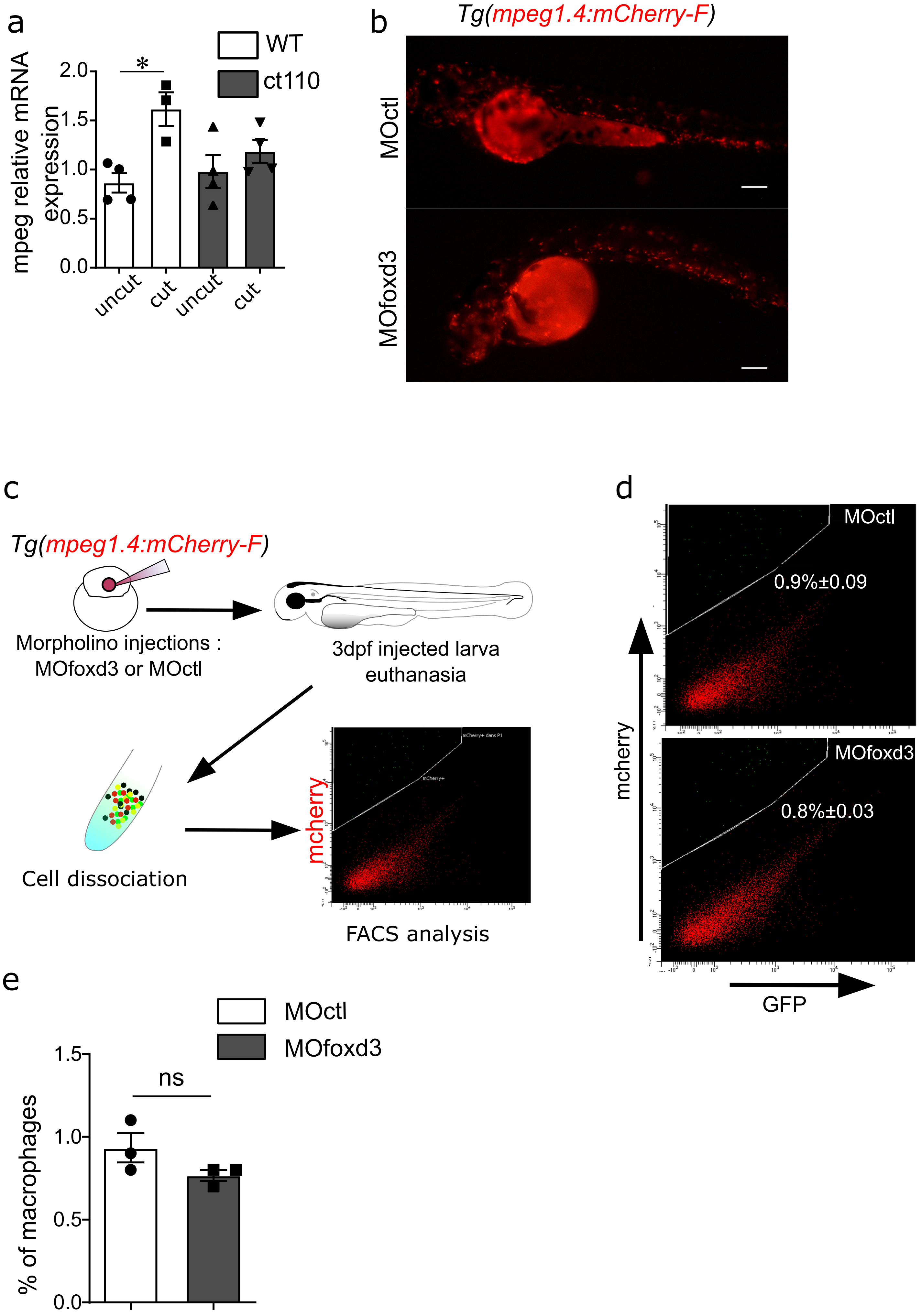
a
WT
ct110
*
mpeg relative mRNA expression
2.0
1.5
1.0
0.5
0.0
uncut
cut
uncut
cut
b
Tg(mpeg1.4:mCherry-F)
MOctl
MOfoxd3
c
Tg(mpeg1.4:mCherry-F)
Morpholino injections :
MOfoxd3 or MOctl
3dpf injected larva
euthanasia
Cell dissociation
mcherry
FACS analysis
d
MOctl
0.9%±0.09
MOfoxd3
0.8%±0.03
mcherry
GFP
e
MOctl
MOfoxd3
ns
% of macrophages
1.5
1.0
0.5
0.0

Supplementary figure 4. **Macrophage response in *foxd3* deficient larvae.** **a** Relative expression of *mpeg* mRNA in intact and amputated WT and *Tg(foxd3:mCherry)^{ct110}* mutant larvae at 24 hpA was assessed by RT-PCR using *ef1a* as reference gene (data are the mean ±SEM, n=15 larvae per groups from 4 independent experiments, one-tailed Mann-Whitney test was performed, p=0.0286 for WT larvae groups, p=0.1714 for mutant larvae groups, *p<0.05). **b** Fluorescence macroscopy images of MO*ctl*- or MO*foxd3*-injected *Tg(mpeg1.4:mCherry-F)* zebrafish larvae (scale bars=400µm). **c** Schematic representation of the experiments, and **d** Representative FACS analysis of mCherry^{+} cells in MO*ctl*-and MO*foxd3*-injected larvae at 3dpf. The white gates represent the mCherry^{+} population and the mean percentage ± SEM of mCherry^{+} cells are indicated. **e** Quantification of the FACS analysis data of *Tg(mpeg1.4:mCherry-F)* larvae injected with MO*ctl* or MO*foxd3* (mean number ± SEM of mCherry^{+} cells, N=150 larvae per groups from 3 independent experiments, two-tailed Mann-Whitney test was performed, p=0.300).

a

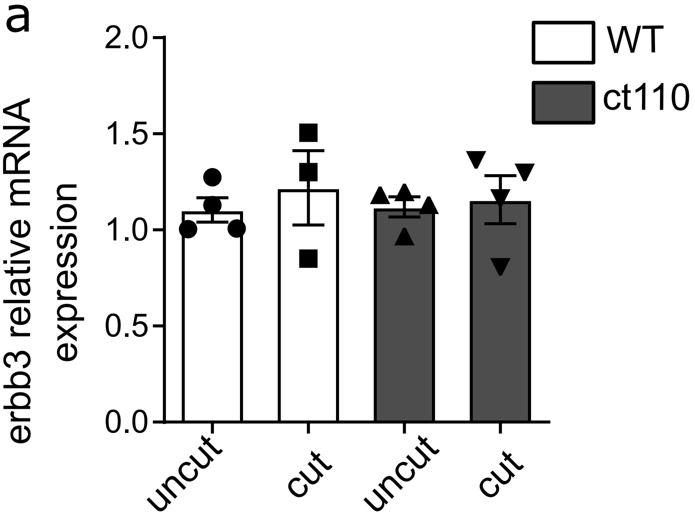

erbb3 relative mRNA expression
2.0
1.5
1.0
0.5
0.0
WT
ct110
uncut
cut
uncut
cut

Supplementary figure 5. ***Errb3* expression profile. a** Relative expression of *erbb3* mRNAs in WT and *Tg(foxd3:mCherry)^{ct110}*mutant larvae at 24hpA. RT-PCR using *ef1a* as reference gene (graph represent means, error bars show the SEM, n=15 larvae per groups from 4 independent experiments).

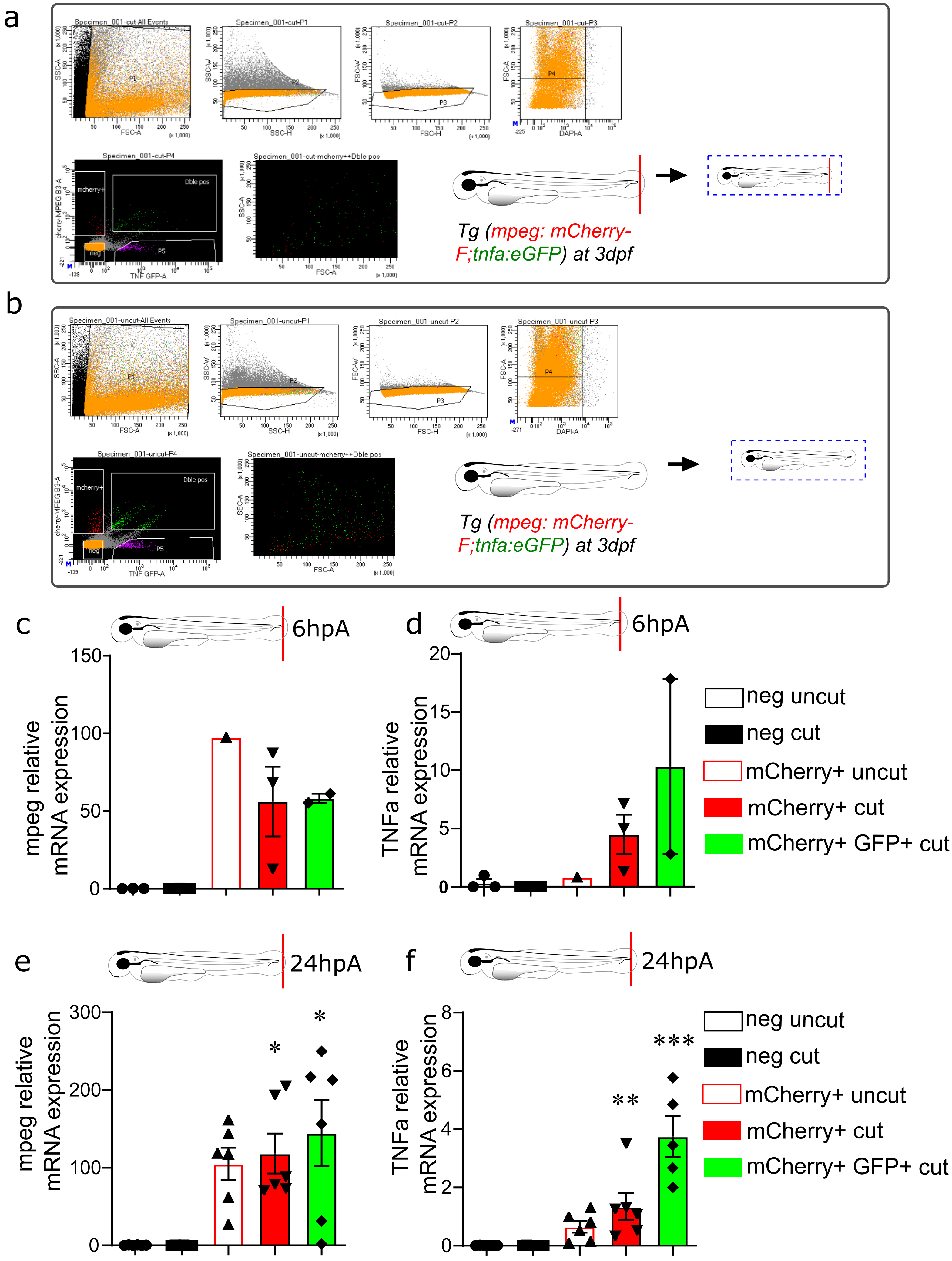
a
Specimen_001-cut-All Events
Specimen_001-cut-P1
Specimen_001-cut-P2
Specimen_001-cut-P3
Specimen_001-cut-P4
Specimen_001-cut-mcherry++Dble pos
Tg (mpeg: mCherry-F;tnfa:eGFP) at 3dpf
b
Specimen_001-uncut-All Events
Specimen_001-uncut-P1
Specimen_001-uncut-P2
Specimen_001-uncut-P3
Specimen_001-uncut-P4
Specimen_001-uncut-mcherry++Dble pos
Tg (mpeg: mCherry-F;tnfa:eGFP) at 3dpf
c
6hpA
mpeg relative mRNA expression
d
6hpA
TNFa relative mRNA expression
neg uncut
neg cut
mCherry+ uncut
mCherry+ cut
mCherry+ GFP+ cut
e
24hpA
mpeg relative mRNA expression
f
24hpA
TNFa relative mRNA expression
neg uncut
neg cut
mCherry+ uncut
mCherry+ cut
mCherry+ GFP+ cut

Supplementary figure 6. **Expression profile of macrophage markers. a, b** Gating strategy of the FACS based cell sorting of *mCherry*$^-$ *GFP*$^-$, *mCherry*$^+$, and *mCherry*$^+$*GFP*$^+$ cells in *Tg(mpeg1:mCherryF/tnfa:eGFP-F)* larvae. Relative expression of **c, e** *mpeg,* and **d, f** *tnfa* in *mCherry*$^-$ *GFP*$^-$, *mCherry*$^+$, and *mCherry*$^+$*GFP*$^+$ cells. Real-time RT-PCR was performed on separated cells using *ef1a* as reference gene. **c, d** Graphs represent the mean value ± SEM, n=200-300 pooled larvae from 4 independent experiments; Two tailed Kruskal Wallis test was performed. **e, f,** Graphs represent the mean value ± SEM, n=200-300 pooled larvae from 6 independent experiments; two-tailed Kruskal-Wallis and Dunn's test for multiple comparisons were performed, *p<0.05,**p<0.001,***p<0.0001.
