# Supplementary material for: NRG1/ErbB signalling controls the dialogue between macrophages and neural crest-derived cells during zebrafish fin regeneration
Source: Nat Commun. 2021 Nov 3;12:6336. doi: 10.1038/s41467-021-26422-5 (PMC8566576; doi:10.1038/s41467-021-26422-5)
Supplement: Supplementary file 3 — Description of Additional Supplementary Files [file 41467_2021_26422_MOESM3_ESM.pdf]

Description of Additional Supplementary Files

Title: Supplementary Data 1.

Description: Table related to Figure 1, showing the lists of the 100 genes upregulated in each cluster of interest compared with the other clusters in the "cut" sample.

Title: Supplementary Data 2.

Description: Lists of the markers used for clustering the different cell types. Each marker combination identifies the cell type.

Title: Supplementary Data 3.

Description: Table related to Figure S1a, showing the list of the 100 genes differentially expressed between "cut" and "uncut" conditions.

Title: Supplementary Data 4.

Description: Table related to Figure S1e-g, showing the lists of the 100 genes upregulated in each cluster of interest compared with the other clusters in the "uncut" sample.

Title: Supplementary Data 5.

Description: Supplementary Table 5. ScRNA-seq quality metrics for "cut" and "uncut" samples. The summary metrics describe the sequencing quality and various features of the detected cells, including mean reads per cell, median genes per cell, number of reads, sequencing saturation percentage, Q30 bases in RNA reads, fraction of total reads assigned to cells, total number of genes detected per sample, median number of UMI counts per cell.

Title: Supplementary Movie 1.

Description: Mesenchymal proliferative cells are dividing in contact with foxd3+ NCdC. Representative time-lapse maximum projections in Tg(foxd3:eGFP/ rcn3:Gal4/UAS:mCherry) larvae of a dividing mesenchymal cell in contact with a foxd3+ NCdC. Image stacks were acquired every 15mins from 6hpA. Arrows indicate successively contacts between the GFP+ cell and mCherry+ cell, and division of the mCherry+ cell. Resolution is 1024 × 1024 pixel using a confocal microscope TCSSP5 SP5 inverted equipped with a HCXPL APO 40×/1.25–0.75 oil objective (Leica). Excitation wavelengths used were 488 nm for EGFP-F and 570 nm for mCherryF. (Scale bar = 8µm)

Title: Supplementary Movie 2.

Description: Foxd3+ NCdC contact preferentially tnfa+ macrophages at the tip of the fin. Representative time-lapse maximum projections in Tg(mpeg1:mCherryF/tnfa:eGFP/foxd3:eGFP). Image stacks were acquired every 15mins from 6hpA. Arrows indicate contacts between macrophages and foxd3+ cells. Time post amputation is shown on top left corner. Resolution is 1024 × 1024 pixel using a confocal microscope TCSSP5 SP5 inverted equipped with a HCXPL APO 40×/1.25–0.75 oil objective (Leica). Excitation wavelengths used were 488 nm for EGFP-F and 570 nm for mCherryF. (Scale bar= 80µm)

Title: Supplementary Movie 3.

Description: Foxd3+ NCdC promote the recruitment of macrophages in the blastema. Representative time-lapse maximum projections in Tg(foxd3:eGFP/mpeg1:mCherry) control morphant. Time post amputation is shown on top left corner. Image stacks were acquired every 15mins from 15minpA. Resolution is 512 ×512 pixel using a confocal microscope TCSSP5 SP5 inverted equipped with a HCXPL APO 40×/1.25–0.75 oil objective (Leica). Excitation wavelengths used were 488 nm for EGFP-F and 570 nm for mCherryF. (Scale bar= 50µm)

Title: Supplementary Movie 4.

Description: Loss of Foxd3+ NCdC impairs the recruitment of macrophages in the blastema. Representative time-lapse maximum projections in Tg(foxd3:eGFP/mpeg1:mCherry) foxd3 morphant. Time post amputation is shown on top left corner. Image stacks were acquired every 15mins from 1hpA. Resolution is 512 ×512 pixel using a confocal microscope TCSSP5 SP5 inverted equipped with a HCXPL APO 40×/1.25–0.75 oil objective (Leica). Excitation wavelengths used were 488 nm for EGFP-F and 570 nm for mCherryF. (Scale bar= 50µm)
