# Supplementary material for: NRG1/ErbB signalling controls the dialogue between macrophages and neural crest-derived cells during zebrafish fin regeneration
Source: Nat Commun. 2021 Nov 3;12:6336. doi: 10.1038/s41467-021-26422-5 (PMC8566576; doi:10.1038/s41467-021-26422-5)
Supplement: Supplementary file 5 — Supplementary Data 2 [file 41467_2021_26422_MOESM5_ESM.pdf]

Supplementary Table 2. **Lists of the markers used for clustering the different cell types.**

Each marker combination identifies the cell type.

**Cluster Epidermis**	**References**	**cluster 2**	**References**	**Cluster Mesenchyme**	**References**	**Cluster Apical epithelial cap**	**Reference**
cldne	[1]	mbpb	[4]	twist1b	[2]	fgf8a	[3]
agr1	[7,8]	lgals1l1	[9]	rcn3		mmp9	[5,6]
krt17	[12]			prrx1a	[10]	mmp13a	[5,11]
oclnb	[15]			col1a1a	[13]	apoeb	[14]
krt4	[15]					mdka	[16]
anxa1c	[17]					mmp2	[5]
anxa1b	[17]					tp63	[18,19]
scel	[20]						

**Cluster Mitotic**	**References**	**Cluster Myeloid cells**	**References**	**Cluster neurons and glia**	**References**
aurkb	[21]	il1b	[22]	irg1l	[23]
mik67	[24]	card9	[25,26]	hmx3a	[27]
top2a	[28]	spi1a	[29]		
cdk1	[30]	mfap4	[31]		
tpx2	[32]	mpeg1.1	[22]		

References

1 Siddiqui, M., Sheikh, H., Tran, C. & Bruce, A. E. The tight junction component Claudin E is required for zebrafish epiboly. *Developmental dynamics : an official publication of the American Association of Anatomists* **239**, 715-722, doi:10.1002/dvdy.22172 (2010).

2 Germanguz, I., Lev, D., Waisman, T., Kim, C. H. & Gitelman, I. Four twist genes in zebrafish, four expression patterns. *Developmental dynamics : an official publication of the American Association of Anatomists* **236**, 2615-2626, doi:10.1002/dvdy.21267 (2007).

3 Kawakami, A., Fukazawa, T. & Takeda, H. Early fin primordia of zebrafish larvae regenerate by a similar growth control mechanism with adult regeneration. *Developmental dynamics : an official publication of the American Association of Anatomists* **231**, 693-699, doi:10.1002/dvdy.20181 (2004).

4 Nawaz, S., Schweitzer, J., Jahn, O. & Werner, H. B. Molecular evolution of myelin basic protein, an abundant structural myelin component. *Glia* **61**, 1364-1377, doi:10.1002/glia.22520 (2013).

5 Andreasen, E. A., Mathew, L. K., Lohr, C. V., Hasson, R. & Tanguay, R. L. Aryl hydrocarbon receptor activation impairs extracellular matrix remodeling during zebra fish fin regeneration. *Toxicological sciences : an official journal of the Society of Toxicology* **95**, 215-226, doi:10.1093/toxsci/kfl119 (2007).

6 Ton, Q. V. & Iovine, M. K. Identification of an evx1-dependent joint-formation pathway during FIN regeneration. *PloS one* **8**, e81240, doi:10.1371/journal.pone.0081240 (2013).

7 Simoes, M. G. *et al.*Denervation impairs regeneration of amputated zebrafish fins. *BMC developmental biology***14**, 780, doi:10.1186/s12861-014-0049-2 (2014).

8 Ivanova, A. S. *et al.* The secreted factor Ag1 missing in higher vertebrates regulates fins regeneration in Danio rerio. *Scientific reports***5**, 8123, doi:10.1038/srep08123 (2015).

9 Ahmed, H., Du, S. J., O'Leary, N. & Vasta, G. R. Biochemical and molecular characterization of galectins from zebrafish (Danio rerio): notochord-specific expression of a prototype galectin during early embryogenesis. *Glycobiology***14**, 219-232, doi:10.1093/glycob/cwh032 (2004).

10 Ocana, O. H. *et al.* Metastatic colonization requires the repression of the epithelial-mesenchymal transition inducer Prrx1. *Cancer cell***22**, 709-724, doi:10.1016/j.ccr.2012.10.012 (2012).

11 Hillegass, J. M., Villano, C. M., Cooper, K. R. & White, L. A. Matrix metalloproteinase-13 is required for zebra fish (Danio rerio) development and is a target for glucocorticoids. *Toxicological sciences : an official journal of the Society of Toxicology***100**, 168-179, doi:10.1093/toxsci/kfm192 (2007).

12 Saxena, S. *et al.* Proteomic analysis of zebrafish caudal fin regeneration. *Mol Cell Proteomics***11**, M111 014118, doi:10.1074/mcp.M111.014118 (2012).

13 Bretaud, S., Nauroy, P., Malbouyres, M. & Ruggiero, F. Fishing for collagen function: About development, regeneration and disease. *Seminars in cell & developmental biology***89**, 100-108, doi:10.1016/j.semcdb.2018.10.002 (2019).

14 Monnot, M. J. *et al.* Epidermal expression of apolipoprotein E gene during fin and scale development and fin regeneration in zebrafish. *Developmental dynamics : an official publication of the American Association of Anatomists***214**, 207-215, doi:10.1002/(SICI)1097-0177(199903)214:3<207::AID-AJA4>3.0.CO;2-5 (1999).

15 Cokus, S. J. *et al.*Tissue-Specific Transcriptomes Reveal Gene Expression Trajectories in Two Maturing Skin Epithelial Layers in Zebrafish Embryos. *G3 (Bethesda)***9**, 3439-3452, doi:10.1534/g3.119.400402 (2019).

16 Schebesta, M., Lien, C. L., Engel, F. B. & Keating, M. T. Transcriptional profiling of caudal fin regeneration in zebrafish. *ScientificWorldJournal***6 Suppl 1**, 38-54, doi:10.1100/tsw.2006.326 (2006).

17 Saxena, S. *et al.* Role of annexin gene and its regulation during zebrafish caudal fin regeneration. *Wound repair and regeneration : official publication of the Wound Healing Society [and] the European Tissue Repair Society***24**, 551-559, doi:10.1111/wrr.12429 (2016).

18 Chen, Y. C. *et al.*Zebrafish Klf4 maintains the ionocyte progenitor population by regulating epidermal stem cell proliferation and lateral inhibition. *PLoS Genet***15**, e1008058, doi:10.1371/journal.pgen.1008058 (2019).

19 Santos-Pereira, J. M., Gallardo-Fuentes, L., Neto, A., Acemel, R. D. & Tena, J. J. Pioneer and repressive functions of p63 during zebrafish embryonic ectoderm specification. *Nature communications***10**, 3049, doi:10.1038/s41467-019-11121-z (2019).

20 Champliaud, M. F. *et al.*Gene characterization of sciellin (SCEL) and protein localization in vertebrate epithelia displaying barrier properties. *Genomics***70**, 264-268, doi:10.1006/geno.2000.6390 (2000).

21 Brown, J. R., Koretke, K. K., Birkeland, M. L., Sanseau, P. & Patrick, D. R. Evolutionary relationships of Aurora kinases: implications for model organism studies and the development of anti-cancer drugs. *BMC Evol Biol***4**, 39, doi:10.1186/1471-2148-4-39 (2004).

22 Nguyen-Chi, M. *et al.* Identification of polarized macrophage subsets in zebrafish. *eLife***4**, e07288, doi:10.7554/eLife.07288 (2015).

23 Van Gennip, J. L. M., Boswell, C. W. & Ciruna, B. Neuroinflammatory signals drive spinal curve formation in zebrafish models of idiopathic scoliosis. *Sci Adv***4**, eaav1781, doi:10.1126/sciadv.aav1781 (2018).

24 Sun, X. & Kaufman, P. D. Ki-67: more than a proliferation marker. *Chromosoma***127**, 175-186, doi:10.1007/s00412-018-0659-8 (2018).

25 Chang, M. X., Chen, W. Q. & Nie, P. Structure and expression pattern of teleost caspase recruitment domain (CARD) containing proteins that are potentially involved in NF-kappaB signalling. *Developmental and comparative immunology***34**, 1-13, doi:10.1016/j.dci.2009.08.002 (2010).

26 Drummond, R. A. *et al.* CARD9(+) microglia promote antifungal immunity via IL-1beta- and CXCL1-mediated neutrophil recruitment. *Nature immunology***20**, 559-570, doi:10.1038/s41590-019-0377-2 (2019).

27 Feng, Y. & Xu, Q. Pivotal role of hmx2 and hmx3 in zebrafish inner ear and lateral line development. *Developmental biology***339**, 507-518, doi:10.1016/j.ydbio.2009.12.028 (2010).

28 Sapetto-Rebow, B. *et al.* Maternal topoisomerase II alpha, not topoisomerase II beta, enables embryonic development of zebrafish top2a-/- mutants. *BMC developmental biology***11**, 71, doi:10.1186/1471-213X-11-71 (2011).

29 Bukrinsky, A., Griffin, K. J., Zhao, Y., Lin, S. & Banerjee, U. Essential role of spi-1-like (spi-1l) in zebrafish myeloid cell differentiation. *Blood* **113**, 2038-2046, doi:10.1182/blood-2008-06-162495 (2009).

30 Gavet, O. & Pines, J. Progressive activation of CyclinB1-Cdk1 coordinates entry to mitosis. *Developmental cell* **18**, 533-543, doi:10.1016/j.devcel.2010.02.013 (2010).

31 Walton, E. M., Cronan, M. R., Beerman, R. W. & Tobin, D. M. The Macrophage-Specific Promoter mfap4 Allows Live, Long-Term Analysis of Macrophage Behavior during Mycobacterial Infection in Zebrafish. *PloS one* **10**, e0138949, doi:10.1371/journal.pone.0138949 (2015).

32 Wadsworth, P. Tpx2. *Curr Biol* **25**, R1156-1158, doi:10.1016/j.cub.2015.10.003 (2015).
