# Supplementary material for: NRG1/ErbB signalling controls the dialogue between macrophages and neural crest-derived cells during zebrafish fin regeneration
Source: Nat Commun. 2021 Nov 3;12:6336. doi: 10.1038/s41467-021-26422-5 (PMC8566576; doi:10.1038/s41467-021-26422-5)
Supplement: Supplementary file 13 — Reporting Summary [file 41467_2021_26422_MOESM13_ESM.pdf]

nature research

Corresponding author(s): Farida Djouad

Last updated by author(s): Jul 26, 2021

Reporting Summary

Nature Research wishes to improve the reproducibility of the work that we publish. This form provides structure for consistency and transparency in reporting. For further information on Nature Research policies, see Authors & Referees and the Editorial Policy Checklist.

Statistics

For all statistical analyses, confirm that the following items are present in the figure legend, table legend, main text, or Methods section.

n/a	Confirmed	
☐	☒	The exact sample size (n) for each experimental group/condition, given as a discrete number and unit of measurement
☐	☒	A statement on whether measurements were taken from distinct samples or whether the same sample was measured repeatedly
☐	☒	The statistical test(s) used AND whether they are one- or two-sided<br>*Only common tests should be described solely by name; describe more complex techniques in the Methods section.*
☐	☒	A description of all covariates tested
☒	☐	A description of any assumptions or corrections, such as tests of normality and adjustment for multiple comparisons
☐	☒	A full description of the statistical parameters including central tendency (e.g. means) or other basic estimates (e.g. regression coefficient) AND variation (e.g. standard deviation) or associated estimates of uncertainty (e.g. confidence intervals)
☐	☒	For null hypothesis testing, the test statistic (e.g. F, t, r) with confidence intervals, effect sizes, degrees of freedom and P value noted<br>*Give P values as exact values whenever suitable.*
☒	☐	For Bayesian analysis, information on the choice of priors and Markov chain Monte Carlo settings
☒	☐	For hierarchical and complex designs, identification of the appropriate level for tests and full reporting of outcomes
☒	☐	Estimates of effect sizes (e.g. Cohen's d, Pearson's r), indicating how they were calculated

Our web collection on statistics for biologists contains articles on many of the points above.

Software and code

Policy information about availability of computer code

Data collection	To collect data of the study, we used:<br>- Leica TCS SP5 : Leica Application Suite V3.2<br>- Leica TCS SP8 X : Leica Application Suite V3.5<br>- Zeiss AxioImager : Zeiss Blue V2.3 – AxioCam MRm
Data analysis	Flowjo software (Tree start, Ashland, Or, USA), GraphPad Prism 6 software (San Diego, CA, USA), Fiji Software Schindelin, J.; Arganda-Carreras, I. & Frise, E. et al. (2012), "Fiji: an open-source platform for biological-image analysis", Nature methods 9(7): 676-682, PMID 22743772, doi:10.1038/nmeth.2019,

For manuscripts utilizing custom algorithms or software that are central to the research but not yet described in published literature, software must be made available to editors/reviewers. We strongly encourage code deposition in a community repository (e.g. GitHub). See the Nature Research guidelines for submitting code & software for further information.

Data

Policy information about availability of data

All manuscripts must include a data availability statement. This statement should provide the following information, where applicable:

- Accession codes, unique identifiers, or web links for publicly available datasets
- A list of figures that have associated raw data
- A description of any restrictions on data availability

All datasets generated during this study were deposited to the Gene Expression Omnibus repository under the series number GSE158851
The scRNA-seq data are deposited in the NCBI Geo database under the series number GSE158851. We created a link for circulation among the selected reviewers to access the private data during the period of manuscript revision. This data will be publicly accessible immediately after publication.

To review GEO accession GSE158851:
Go to https://www.ncbi.nlm.nih.gov/geo/query/acc.cgi?acc=GSE158851

Enter token klkrmycmnhcfpaj into the box

Field-specific reporting

Please select the one below that is the best fit for your research. If you are not sure, read the appropriate sections before making your selection.

☒ Life sciences ☐ Behavioural & social sciences ☐ Ecological, evolutionary & environmental sciences

For a reference copy of the document with all sections, see nature.com/documents/nr-reporting-summary-flat.pdf

Life sciences study design

All studies must disclose on these points even when the disclosure is negative.

Sample size	Sample sizes estimation was based on previous studies : https://pubmed.ncbi.nlm.nih.gov/26154973/ https://pubmed.ncbi.nlm.nih.gov/28796253/
Data exclusions	No data were excluded.
Replication	For RTqPCR : at least three experiments per conditions with a pool of at least 15 samples, were conducted independantly.<br>For FACS experiments : at least 3 experiments were conducted independantly.
Randomization	Morpholino injections were performed with a mixed pool of embryos from different parents randomly, then the embryos were assigned randomly to each group (time points) during the duration of the experiments (RTqPCR, PH3 detection, lenght measurments). Allocation of mutants ct110r to the different time point groups was performed randomly.
Blinding	Data were collected by one or two exprimentators (BL-B, AB, CB) and the analysis was performed by different experimentators (BL-B, CB, SA, AB).

Reporting for specific materials, systems and methods

We require information from authors about some types of materials, experimental systems and methods used in many studies. Here, indicate whether each material, system or method listed is relevant to your study. If you are not sure if a list item applies to your research, read the appropriate section before selecting a response.

Materials & experimental systems

n/a	Involved in the study	
☐	☒	Antibodies
☒	☐	Eukaryotic cell lines
☒	☐	Palaeontology
☐	☒	Animals and other organisms
☒	☐	Human research participants
☒	☐	Clinical data

Methods

n/a	Involved in the study	
☒	☐	ChIP-seq
☐	☒	Flow cytometry
☒	☐	MRI-based neuroimaging

Antibodies

Antibodies used	anti-phosphorylated histone 3 antibody (Cell Signaling, ref 9701, dilution: 1/500).
Validation	https://www.cellsignal.com/products/primary-antibodies/phospho-histone-h3-ser10-antibody/9701<br>https://pubmed.ncbi.nlm.nih.gov/24046323/<br>https://pubmed.ncbi.nlm.nih.gov/25344692/<br>https://pubmed.ncbi.nlm.nih.gov/28796253/<br>https://pubmed.ncbi.nlm.nih.gov/26154973/

Animals and other organisms

Policy information about studies involving animals; ARRIVE guidelines recommended for reporting animal research

Laboratory animals	Experiments were performed using the AB zebrafish stain (ZIRC), and the transgenic line Tg(mpeg1:mCherry-F) to visualize macrophages, Tg(tnfa :eGFP-F) to visualize tnfa expression Tg(rcn3:gal4/UAS:DsRed) to visualize mesenchymal cells Tg(foxd3:eGFP-F) 55 and Tg(sox10:eGFP-F) to visualize NC cells, Tg(col2a:mCherry) to visualize chondrocytes. Homozygous larvae from the Tg(foxd3:mCherry)ct110 line were used as Foxd3 mutants . Embryos were obtained from adult fish pairs by natural spawning.

Wild animals | The study did not involve wild animals.

Field-collected samples | The study did not involve samples collected from the field.

Ethics oversight | All animal experiments described in this study were carried out at the University of Montpellier according to the European Union guidelines for the handling of laboratory animals (http://ec.europa.eu/environment/chemicals/lab_animals/home_en.htm) and were approved by the Direction Sanitaire et Vétérinaire de l'Hérault and Comité d'Ethique pour l'Expérimentation Animale under reference CEEA-LR-13007.

Note that full information on the approval of the study protocol must also be provided in the manuscript.

Flow Cytometry

Plots

Confirm that:

☒ The axis labels state the marker and fluorochrome used (e.g. CD4-FITC).

☒ The axis scales are clearly visible. Include numbers along axes only for bottom left plot of group (a 'group' is an analysis of identical markers).

☒ All plots are contour plots with outliers or pseudocolor plots.

☒ A numerical value for number of cells or percentage (with statistics) is provided.

Methodology

Sample preparation | 200-300 Tg(tnfa:eGFP-F/mpeg1:mCherryF) larvae were either amputated , rinsed with PBS, and digest using FACSmax. Then passed thgrough a 40-µm cell strainer (Falcon, France). Isolated cells were washed in PBS/2 mM ethylenediaminetetraacetic acid (EDTA)/2% Foetal Calf Serum (FCS), filtered through a 40-µm cell strainer and centrifugated for 5mins at 300g.

Instrument | Counting of mCherry+eGFP− and mCherry+eGFP+ and negative cells was performed on LSRFortessa (BD Bioscience, France). Cell sorting was performed using FACS ARIA (BD Bioscience, France) and collected in 50% FCS/50% Leibovitz L-15 medium (21083-027, Gibco, France) on ice.

Software | Data were analyzed using the Flowjo software (Tree start, Ashland, Or, USA).

Cell population abundance | The relevant cell populations within the post-sort fractions were very small. However, we evaluated the purity of these populations by RT-qPCR using specific primers for mpeg1 and tnfa to quantify the expression levels of total macrophage and pro-inflammatory macrophage markers, respectively, in the relevant populations.

Gating strategy | We first performed a FSC/SSC gating avoiding small particles with high SSC. Then, we selected the single cell events (FSC-H/FSC-A) to finally gate tnfa and mpeg1 positive cells.

☒ Tick this box to confirm that a figure exemplifying the gating strategy is provided in the Supplementary Information.
